# Supplementation with Sodium Selenite and Selenium-Enriched Microalgae Biomass Show Varying Effects on Blood Enzymes Activities, Antioxidant Response, and Accumulation in Common Barbel (*Barbus barbus*)

**DOI:** 10.1155/2014/408270

**Published:** 2014-02-26

**Authors:** Antonín Kouba, Josef Velíšek, Alžběta Stará, Jiří Masojídek, Pavel Kozák

**Affiliations:** ^1^Research Institute of Fish Culture and Hydrobiology, South Bohemian Research Center of Aquaculture and Biodiversity of Hydrocenoses, Faculty of Fisheries and Protection of Waters, University of South Bohemia in České Budějovice, Zátiší 728/II, 389 25 Vodňany, Czech Republic; ^2^Department of Phototrophic Microorganisms, AlgaTech, Institute of Microbiology, Academy of Sciences, Opatovický mlýn, 379 81 Třeboň, Czech Republic

## Abstract

Yearling common barbel (*Barbus barbus* L.) were fed four purified casein-based diets for 6 weeks in outdoor cages. Besides control diet, these were supplemented with 0.3 mg kg^−1^ dw selenium (Se) from sodium selenite, or 0.3 and 1.0 mg kg^−1^ from Se-enriched microalgae biomass (*Chlorella*), a previously untested Se source for fish. Fish mortality, growth, Se accumulation in muscle and liver, and activity of selected enzymes in blood plasma, muscle, liver, and intestine were evaluated. There was no mortality, and no differences in fish growth, among groups. Se concentrations in muscle and liver, activity of alanine aminotransferase and creatine kinase in blood plasma, glutathione reductase (GR) in muscle, and GR and catalase in muscle and liver suggested that selenium from Se-enriched *Chlorella* is more readily accumulated and biologically active while being less toxic than sodium selenite.

## 1. Introduction

Selenium (Se) is a trace element essential for all living organisms, where it is a constituent of selenoproteins [1, 2]. In aquaculture, it is used as a supplement to live feed, improving their nutritional profile to fulfil requirements of cultured fish [3–5]. Elevation of dietary Se helps fish to withstand stress [6, 7] and moderates toxicity of heavy metals such as mercury and cadmium [8, 9]. Selenium contributes to stabilizing meat quality of farmed animals [10, 11]. Studies on humans show its anticancer properties [12, 13] and the positive effects on reproduction are well known [14, 15]. However, selenium-related studies also confirm that the concentration range in which Se is considered essential or toxic is quite narrow (see further). Various forms of Se supplementation (selenite, selenate, selenomethionine (Se-Met), selenocysteine (Se-Cys), and Se-enriched yeasts) have been studied in fish species including rainbow trout *Oncorhynchus mykiss* [16], Atlantic salmon *Salmo salar* [17, 18], channel catfish *Ictalurus punctatus* [19], hybrid striped bass *Morone chrysops *× *M*. *saxatilis* [20], common carp *Cyprinus carpio* [21], and crucian carp *Carassius auratus gibelio* [22]. Although Zhou et al. [23] confirmed that Se nanoparticles are more effective in increasing muscle Se content compared to Se-Met in the crucian carp, studies mentioned above confirmed that organic forms are more digestible, better accumulated, and more biologically active, while being less toxic than inorganic forms. Recently, supplementation of Se-enriched garlic, primarily containing selenium as *γ*-gluthamyl selenomethylselenocysteine and selenomethylselenocysteine, has been proposed as an alternative in producing Se-enriched meat of African catfish [24, 25]. However, Se-enriched yeasts are currently the supplement of choice.

Feeding microalgae to fish has been used successfully to provide an important natural source of bioactive compounds, such as carotenoids, fatty acids, polysaccharides, amino acids, and vitamins [26]. Progress in microalgae cultivation has included development of fed-batch phototrophic cultivation of the Se-enriched microalgae (Masojídek et al., unpublished data). This source of Se has not been evaluated as a fish dietary supplement, so we compared Se-enriched microalgae biomass and sodium selenite as dietary supplements for indoor culture of common barbel (*Barbus barbus* L.) with respect to effects on fish growth, Se accumulation, and the activity of selected enzymes in certain tissues.

## 2. Materials and Methods

### 2.1. Diets

Experimental purified casein-based diets followed formulation of Wang et al. [22] with some modifications including the use of rapeseed oil and the commercially available vitamin/mineral premix (Roboran H, Univit Ltd.) with 8.39 g kg^−1^ replaced with microalgae *Chlorella* spp. (cf. *C. vulgaris* Beijerinck) biomass (Table 1). Experimental diets were supplemented with sodium selenite or Se-enriched microalgae to obtain Se concentrations of 0.3 mg kg^−1^ dry weight (dw) from sodium selenite (Se 0.3) and 0.3 and 1.0 mg kg^−1^ from Se-enriched microalgae biomass, (Algadiet 0.3 and Algadiet 1.0) (Table 2). A control diet contained Se-free microalgae biomass. Chemical analysis showed Se concentrations of 0.06, 0.29, 0.31, and 1.04 mg kg^−1^ dw in control, Se 0.3, Algadiet 0.3, and Algadiet 1.0 diet, respectively. An Se level near 0.3 mg kg^−1^ in the diet has been stated as a requirement for various fish species [1, 27], while a concentration of 1.0 mg kg^−1^ may be associated with the onset of oxidative damage leading to impaired antioxidant status [28, 29].

Components of artificial diets were sourced as follows: casein, gelatine, and dextrin were purchased from VWR International (Radnor, PA, USA). Cellulose and carboxymethyl cellulose were obtained from Alfa Aesar GmbH & Co KG (Karlsruhe, Germany). Microalgae were cultivated at the Institute of Microbiology in Třeboň, Czech Republic. Pure sodium selenite was supplied by Sigma-Aldrich (St. Louis, MO, USA). Ingredients were mixed in a commercial food mixer. Oil was gradually added during mixing. Water was slowly blended into the mix to obtain dough of suitable texture for processing through a cold extruder. Obtained spaghetti-like strips were manually cut, dried in a household fruit dryer at 60°C for 24 h, and frozen at −20°C until feeding. The composition of the diets (Table 1) was analyzed in the accredited laboratory of the State Veterinary Institute in Prague, Czech Republic.

### 2.2. Animals and Husbandry

Common barbel (*Barbus barbus* L.) yearlings weighing 48.4 ± 6.1 g (range 40.0–70.7 g) were stocked in outdoor experimental feeding cages (60 × 40 × 30 cm) supplied with microfiltrated (109 **μ**m pore size) water from the Blanice River, South Bohemia. Initial standard body length and weight of stocked fish (Table 3) were not significantly different among groups (*F* = 1.03, *P* = 0.381, and *F* = 0.16, *P* = 0.922, resp.). Groups of 10 fish were acclimatized and fed the control diet for 10 days prior to the experimental period of 6 weeks. Fish were provided four equal feedings per day (08:00, 12:00, 15:00, and 18:00 h), initially at a rate of 1.5% of live weight to ensure that all feed was rapidly consumed. Feeding rate was adjusted according to batch weighing of fish every two weeks. Fish were not fed on the final day before sampling at the end of the experiment. Water temperature (19.2 ± 1.5°C) was registered hourly using Minikin loggers (Environmental Measuring Systems, Brno, Czech Republic). Dissolved oxygen concentration (8.5 ± 0.8 mg L^−1^) and pH (7.5 ± 0.1) were measured daily by means of oximeter Oxi 330i and pH meter pH/Cond 340i (WTW GmbH, Weilheim, Germany), respectively. The trial was conducted in triplicate.

### 2.3. Sampling Protocol

At the end of the trial, fish were measured to the nearest 0.1 mm (standard length measured from the tip of the head to the base hypural plate at caudal flexion) and weighed to the nearest 1.0 g. Eight randomly selected fish from each replicate were sacrificed (stunned by a blow with a blunt object over the head) and sampled for biochemical analysis. The study was conducted according to the principles of the Ethical Committee for the Protection of Animals in Research of the University of South Bohemia, Faculty of Fisheries and Protection of Waters, Research Institute of Fish Culture and Hydrobiology, Vodňany, based on the EU harmonized animal welfare act of Czech Republic. The principles of laboratory animal care and the national laws 246/1992 and regulations on animal welfare were followed (Ref. number 22761/2009-17210).

### 2.4. Biochemical Blood Plasma Indices

Blood was drawn from the *vena caudalis* and samples stabilized with 40 IU sodium heparin per 1 mL. Blood was separated by centrifugation at 12 000 ×g for 10 min at 4°C, and plasma samples were held at −80°C until analysis. The following biochemical indices were evaluated: aspartate aminotransferase (AST), alanine aminotransferase (ALT), lactate dehydrogenase (LDH), and creatine kinase (CK). For the biochemical analysis of plasma, the VETTEST 8008 analyzer (IDEXX Laboratories Inc., USA) was used.

### 2.5. Tissue Samples and Preparation of Postmitochondrial Supernatant

After blood sampling, the muscle, liver, and intestine were quickly dissected, immediately frozen, and stored at −80°C. For analysis, frozen tissue samples were weighed and homogenized (1 : 10, w/v) with an Ultra Turrax homogenizer (Ika, Germany) using 50 mM potassium phosphate buffer, pH 7.0, containing 0.5 mM EDTA. The homogenate was divided into two portions, one for measuring thiobarbituric acid reactive substances (TBARS) and a second was centrifuged at 12 000 ×g for 30 min at 4°C to obtain the postmitochondrial supernatant for other antioxidant parameter analyses.

### 2.6. Indices of Oxidative Stress

The TBARS method was used to evaluate lipid peroxidation [30]. The concentration of TBARS was calculated by the absorption at 535 nm and a molar extinction coefficient of 156 mM cm^−1^. The value was expressed as nanomoles of TBARS per mg of wet tissue.

### 2.7. Antioxidant Parameters

Total superoxide dismutase (SOD; EC 1.15.1.1) activity was determined by the method of S. Marklund and G. Marklund [31]. This assay depends on the autoxidation of pyrogallol. Superoxide dismutase activity was assessed spectrophotometrically at 420 nm and expressed as the amount of enzyme per milligram of protein. The catalase (CAT; EC 1.11.1.6) activity was assayed spectrophotometrically as H_2_O_2_ breakdown at 240 nm [32]. Glutathione peroxidase (GPx; EC 1.11.1.9) activity was calculated from the rate of NADPH oxidation at 340 nm in the reaction with glutathione reductase (GR; EC 1.6.4.2). The specific activity was determined using the extinction coefficient 6.22 mM cm^−1^ [33]. Glutathione reductase activity was determined spectrophotometrically, measuring NADPH oxidation at 340 nm [34]. One unit of CAT, GPx, or GR activity is defined as the amount of enzyme that consumes 1 mol L^−1^ of substrate or generates 1 mol L^−1^ of product per min expressed in international units per mg of protein.

### 2.8. Protein Estimation

Protein levels were estimated spectrophotometrically (Tecan Infinite M200, Schoeller Instruments, Prague, Czech Republic) using bovine serum albumin as a standard [35].

### 2.9. Selenium Concentration

Concentration of selenium was determined in the microalgae biomass, experimental diets, and fish tissues by means of ICP-MS analysis. Muscle of four randomly selected individuals per replication was analysed. Insufficient liver tissue for analysis required that we pair target tissue from two randomly chosen fish (final *n* = 4 per replication). The detection limit of Se was 0.05 mg kg^−1^ dw in all matrices.

### 2.10. Statistical Analysis

Statistical analysis was carried out using Statistica software 9.0 for Windows (StatSoft, Czech Republic). Data were first tested for normality and homoscedasticity with Kolmogorov-Smirnov and Levene's tests, respectively. If those conditions were satisfied, one-way analysis of variance (ANOVA) was employed to reveal significant differences in measured variables among control and experimental groups. When a difference was detected (*P* < 0.05), Tukey's multiple comparison test was applied to identify which treatments were significantly different. If the conditions for ANOVA were not satisfied, the nonparametric Kruskal-Wallis test followed by multiple comparisons of mean ranks for all groups was used [36]. All data are expressed as means ± s.d.

## 3. Results

No mortality was observed during the trial. Mean standard body length was 16.1 to 16.5 mm and weight was 52.1 to 54.6 g, at the end of the experiment (Table 3). Values were not significantly different (*F* = 1.47, *P* = 0.228, and *F* = 0.93, *P* = 0.428, resp.), and no clear trend of treatments was observed.

The final Se concentration in muscle of fish from the control group was 0.71 ± 0.08 mg kg^−1^ dw. A concentration of 0.80 ± 0.10 and 0.88 ± 0.09 mg kg^−1^ dw was found in the muscle of fish fed Se 0.3 and Algadiet 0.3, respectively, with the latter being significantly higher than that of the control (*P* < 0.002). The Algadiet 1.0 group showed Se concentration of 1.18 ± 0.12 mg kg^−1^ dw, significantly higher than other groups (*P* < 10^−3^ in all cases). Similar trends with greater mean concentrations of Se were found in liver, 2.22, 2.92, 3.18, and 3.71 mg kg^−1^ dw in the control, Se 0.3, Algadiet 0.3, and 1.0 groups, respectively.

Activity of ALT and CK was significantly (*P* < 0.01) greater in the Se 0.3 and Algadiet 1.0 groups as compared to the control group. The AST and LDH activity was similar in all groups (Table 4).

Among the test groups, none showed significant differences from the control in TBARS level, GPx, or SOD activity in muscle, liver, or intestine (Table 5).

The activity of CAT in liver was significantly (*P* < 0.01) higher than the in the control in all Se-supplemented groups. In case of muscle, higher CAT activities were seen in the Se 0.3 and Algadiet 1.0 groups (Table 5) compared to the control. No differences were found in CAT activity in intestine. Significantly lower (*P* < 0.01) muscle GR activity was observed in all treatment groups compared to the control. GR activity observed in liver or intestine did not differ from the enzyme activity in the control group.

## 4. Discussion

Selenium is an important micronutrient in animals as well as in humans [37, 38]. Its deficiency has various negative impacts [8, 39, 40]; however, the line between Se nutritive requirements and toxicity is narrow [41–44]. Research has focussed on comparing doses and forms of Se supplementation. Studies on fish have confirmed that organic forms of Se are more digestible, better accumulated in tissue, and more biologically active than inorganic [6, 19, 21, 22]. In our experiment using Se-enriched microalgae biomass as feed supplement for common barbel, no significant differences in final fish size were found. However, Se concentration in muscle and liver of Se 0.3 and Algadiet 0.3 supplemented groups suggested that Se from Se-enriched microalgae is more readily accumulated in tissue.

Recently, Se-enriched yeasts containing 54–74% total Se in the form of selenomethionine (Se-Met) [45] have been promoted. This form of selenium is considered to facilitate adequate digestibility and consequent biological activity, since Se-Met is directly incorporated into protein in place of methionine (Met), as tRNA^Met^ does not discriminate between Met and Se-Met [46]. Reports of content of Se-Met in *Chlorella* biomass are conflicting. De Alcantara et al. [47] assumed intracellularly fixed selenium in the *Chlorella* biomass to be Se-Met. More than 70% of protein-bound selenium has been found to be Se-Met, although this constituted only a minor fraction (0.7%) of the total Se in the *Chlorella* biomass according to Fan et al. [48]. Dimethylselenonium propionate has been reported as the principal Se-containing compound in *Chlorella* [49]. Neumann et al. [50] found 24–39% of total selenium to be in the form of Se-Met when incubating *Chlorella* in differing mineral-nutrient solutions. However, content of selenocysteine (Se-Cys) was higher, at 48 to 76%. Differing Se compounds in Se-enriched yeasts and Se-enriched microalgae might suggest using the former, as Se-Met is probably the most active compound. Se-enriched microalgae may, however, benefit from the presence of specific bioactive compounds such as antioxidants, pigments, fatty acids, polysaccharides, and immunoactive substances.

Blood enzyme levels provide vital information to aid in fish health assessment [51, 52]. Biochemical alterations are usually the first detectable and quantifiable responses to environmental change. Alteration of blood biochemical composition might be indicative of unsuitable environmental conditions or the presence of stress factors such as toxic chemicals, overcrowding, and some common aquaculture procedures [53, 54]. We analysed LDH, CK, and the transaminases ALT and AST on the basis that alterations in the activity of these enzymes might be stress induced and correspond to tissue damage. Increased ALT and CK activity indicates the induction of transamination processes that occur as amino acid input into the tricarboxylic acid cycle. In this way, fish may have coped with the energy deficit during stress associated with the inorganic Se 0.3 and organic Algadiet 1.0 treatments. Differences in activity of ALT and CK in groups with comparable selenium concentrations (Se 0.3 and Algadiet 0.3) suggest higher toxicity of the inorganic form. Reports concerning effects of selenium on fish blood biochemical profile are scarce. Increased activity of AST and ALT was found in African catfish fed 0.5 g kg^−1^ Se-enriched yeasts, suggesting that high Se levels in the diet exhibit a toxic effect related to liver and kidney dysfunction [55].

Selenium is essential for the proper functioning of the antioxidant enzymes, which protect against oxidative stress [56, 57]. Maintaining cellular oxidative homeostasis involves the antioxidant defence system, including enzymes such as CAT, GR, SOD, and GPx, which can be employed as biomarkers of oxidative stress [29, 40, 58]. In this study, the CAT activity in liver was significantly higher in all Se-supplemented groups, but in muscle was higher than the control group only in the Se 0.3 and Algadiet 1.0 groups. CAT activity prevents adverse effects of oxidative stress in cells. This enzyme is mainly located in the peroxisomes and is responsible for the reduction of hydrogen peroxide produced in the metabolic pathways of long-chain fatty acids [59]. Increased CAT activity may have indicated that its antioxidant defence was exhausted by the level of hydroperoxide products, reflecting a possible failure of the antioxidant system caused by the Se 0.3 and the higher dose of organic Se in the Algadiet 1.0 group. Increased CAT activity associated with elevated dietary selenium has been reported in various fish [21, 28, 29] as well as crustaceans [60–62].

Glutathione reductase activity maintains the cytosolic concentration of reduced glutathione [63, 64]. Significantly (*P* < 0.01) lower muscle GR activity was observed in all Se-supplemented groups compared to the control. Decreased GR activity may lead to the depletion of glutathione if its loss cannot be compensated for by the synthesis of new glutathione molecules [65]. Lower muscle GR activity has also been observed in coho salmon [40], largemouth bass [66], tench [29], and red swamp crayfish *Procambarus clarkii* [62, 67].

Results of the present study support the hypothesis that alteration in activity of CAT and GR enzymes in muscle and liver can counteract the prooxidant effect induced by elevated dietary Se. Oxidative stress indicators in muscle and liver of inorganic Se and higher concentration of organic Se-treated common barbel suggest that these tissues may possess differing abilities to counteract Se-prooxidant effects, with muscle showing a higher level of oxidative stress. Se supplementation at levels equal to or higher than those employed in this study (Se 0.3 and Algadiet 1.0) may accelerate the antioxidant condition of barbell, and, therefore, the optimal Se dietary concentration may be lower in this species.

## 5. Conclusion

Selenium concentrations and enzyme activity in tissues of common barbel suggest that supplementation with Se-enriched *Chlorella* biomass is more effective than inorganic sodium selenite. Direct comparison of Se-enriched microalgae with organic forms (especially Se-Met) and Se-enriched yeasts is needed. Efficacy of Se-enriched microalgae when compared with Se-enriched yeasts may be lower in commonly investigated indices. Use of Se-enriched microalgae biomass in aquaculture feedstuffs could be supported by the presence of specific bioactive compounds like polyunsaturated fatty acids, pigments, and vitamins.

## Figures and Tables

**Table 1:** Formulation, mean approximate composition, and gross energy of experimental diets.

Ingredients (g kg^−1^)	
Casein	320.0
Gelatine	80.0
Dextrine	280.0
Cellulose	190.0
Rapeseed oil	60.0
Carboxymethyl cellulose	20.0
Vitamin and mineral premix^a^	41.61
Microalgae biomass^b^	8.39

Proximate composition (g kg^−1^ dw)	
Dry matter	888.1
Crude protein	336.4
NFE^c^	474.6
Crude lipid	21.4
Crude fibre	139.6
Ash	28.0
Gross energy (MJ kg^−1^)^d^	16.9

^a^Composition of the vitamin and mineral premix (Roboran H) provided by the manufacturer: vitamin A, 500 000 IU; vitamin D3, 100 000 IU; vitamin K, 20 mg kg^−1^; vitamin E, 750 mg kg^−1^; vitamin B1, 25 mg kg^−1^; vitamin B2, 40 mg kg^−1^; vitamin B6, 20 mg kg^−1^; vitamin B12, 0.5 mg kg^−1^; niacin, 200 mg kg^−1^; calcium pantothenate, 200 mg kg^−1^; choline chloride, 20 000 mg kg^−1^; biotin, 10 mg kg^−1^; cobalt as CoSO_4_·7H_2_O 6 mg kg^−1^; copper as CuO 150 mg kg^−1^; iodine as KI 20 mg kg^−1^; iron as FeSO_4_·H_2_O 380 mg kg^−1^; manganese as MnO 110 mg kg^−1^; zinc as ZnO 140 mg kg^−1^.

^b ^Composition of Se-free and Se-enriched algal biomass differed among treatments (see Table 2).

^c ^Nitrogen-free extract, NFE = 1000 − (protein + lipid + ash + crude fibre) (g kg^−1^).

^d ^Calculated assuming conversion factors of 23.6, 39.5, and 17.2 MJ kg^−1^ for protein, lipid, and NFE, respectively [68].

**Table 2:** Sodium selenite, Se-free and/or Se-enriched microalgae supplement, and calculated concentration of selenium (mg kg^−1^ dw) in experimental diets.

Se source/treatment	Control	Se 0.3	Algadiet 0.3	Algadiet 1.0
Sodium selenite	−	+	−	−
Se-free^a^/Se-enriched^b^ microalgae biomass ratio (%)	100/0	100/0	30/70	0/100
Calculated additive concentration	0.0	0.3	0.3	1.0

^a^Se concentration below detection limit of 0.05 mg kg^−1^dw.

^b ^Se concentration of 119.2 mg kg^−1^ dw.

**Table 3:** Initial and final size indices of common barbel (*Barbus barbus* L.) yearlings fed on control diet containing Se-free microalgae biomass and diets supplemented by either sodium selenite or Se-enriched microalgae (replacing Se-free microalgae) to obtain Se concentrations of 0.3 mg kg^−1 ^from sodium selenite (Se 0.3) or 0.3 and 1.0 mg kg^−1 ^from Se-enriched microalgae biomass, Algadiet 0.3 and Algadiet 1.0, respectively.

Growth indices/treatment	Control	Se 0.3	Algadiet 0.3	Algadiet 1.0
Initial standard body length (mm)	14.2 ± 0.7	14.3 ± 0.8	14.4 ± 0.6	14.4 ± 0.6
Initial weight (g)	47.8 ± 6.3	48.5 ± 6.9	48.9 ± 5.9	48.3 ± 5.5
Final standard body length (mm)	16.1 ± 0.9	16.3 ± 0.7	16.3 ± 0.8	16.5 ± 0.7
Final weight (g)	54.2 ± 6.9	52.1 ± 6.6	52.4 ± 5.5	54.6 ± 7.5

**Table 4:** Blood enzymes activity in common barbel (*Barbus barbus* L.) yearlings fed on control diet containing Se-free microalgae biomass and diets supplemented by either sodium selenite or Se-enriched microalgae (replacing Se-free microalgae) to obtain Se concentrations of 0.3 mg kg^−1^from sodium selenite (Se 0.3) or 0.3 and 1.0 mg kg^−1^from Se-enriched microalgae biomass Algadiet 0.3 and Algadiet 1.0, respectively.

Indices	Test groups			
	Control	Se 0.3	Algadiet 0.3	Algadiet 1.0
AST (**μ**kat L^−1^)	22.2 ± 5.6^a^	23.6 ± 5.0^a^	20.9 ± 5.1^a^	19.7 ± 4.6^a^
ALT (**μ**kat L^−1^)	1.9 ± 0.7^a^	2.6 ± 0.9^b^	2.2 ± 0.8^ab^	3.3 ± 0.7^c^
LDH (**μ**kat L^−1^)	32.0 ± 7.6^a^	34.6 ± 7.0^a^	31.4 ± 8.3^a^	29.0 ± 6.5^a^
CK (**μ**kat L^−1^)	35.2 ± 8.6^a^	42.5 ± 7.6^b^	30.6 ± 6.4^a^	42.2 ± 6.7^b^

Different superscripts in the same row indicate significance difference *α* = 0.01; *n* = 8 per replication.

**Table 5:** Oxidative stress and antioxidant responses in tissue of common barbel (*Barbus barbus* L.) yearlings fed on control diet containing Se-free microalgae biomass and on diets supplemented by either sodium selenite or Se-enriched microalgae (replacing Se-free microalgae) to obtain Se concentrations of 0.3 mg kg^−1^from sodium selenite (Se 0.3) or 0.3 and 1.0 mg kg^−1^from Se-enriched microalgae biomass Algadiet 0.3 and Algadiet 1.0, respectively.

Indices	Tissues	Test groups			
		Control	Se 0.3	Algadiet 0.3	Algadiet 1.0
TBARS (nmol g ww^−1^)	Muscle	8.4 ± 3.8^a^	7.5 ± 4.4^a^	7.1 ± 3.6^a^	6.5 ± 3.6^a^
	Liver	32.8 ± 9.1^a^	33.3 ± 8.3^a^	32.3 ± 9.8^a^	31.4 ± 8.1^a^
	Intestine	3.4 ± 1.1^a^	3.1 ± 0.9^a^	2.9 ± 0.8^a^	2.8 ± 0.9^a^

SOD (U mg^−1^ protein)	Muscle	21.9 ± 4.5^a^	19.3 ± 5.4^a^	20.5 ± 5.4^a^	21.4 ± 4.2^a^
	Liver	141.9 ± 21.8^a^	127.1 ± 21.2^a^	137.0 ± 15.3^a^	164.0 ± 26.3^a^
	Intestine	5.1 ± 1.6^a^	4.8 ± 1.1^a^	5.0 ± 1.3^a^	4.3 ± 1.2^a^

CAT (mU mg^−1^ protein)	Muscle	2.4 ± 0.8^a^	3.7 ± 0.8^b^	2.7 ± 1.0^a^	4.6 ± 1.1^c^
	Liver	26.9 ± 8.1^a^	41.7 ± 8.6^bc^	35.4 ± 7.7^b^	47.1 ± 9.6^c^
	Intestine	0.8 ± 0.3^a^	0.9 ± 0.3^a^	0.8 ± 0.2^a^	0.8 ± 0.2^a^

GPx (U mg^−1^ protein)	Muscle	66.4 ± 14.9^a^	63.0 ± 10.7^a^	64.8 ± 9.4^a^	62.5 ± 8.5^a^
	Liver	194.4 ± 45.1^a^	185.6 ± 35.4^a^	177.4 ± 32.9^a^	198.2 ± 26.0^a^
	Intestine	29.6 ± 6.6^a^	29.5 ± 7.6^a^	27.9 ± 6.4^a^	25.0 ± 6.3^a^

GR (U mg^−1^ protein)	Muscle	17.4 ± 7.3^b^	7.3 ± 3.2^a^	10.0 ± 3.6^a^	6.8 ± 2.8^a^
	Liver	6.0 ± 2.3^a^	6.9 ± 2.0^a^	6.2 ± 2.3^a^	6.0 ± 2.6^a^
	Intestine	8.1 ± 2.0^a^	7.7 ± 2.7^a^	7.1 ± 2.6^a^	6.7 ± 2.5^a^

Different superscripts in the same row indicate significant differences *α* = 0.01; *n* = 8 per replication.
